# Supplementary material for: Causal Relationship between Aging and Anorexia Nervosa: A White-Matter-Microstructure-Mediated Mendelian Randomization Analysis
Source: Biomedicines. 2024 Aug 16;12(8):1874. doi: 10.3390/biomedicines12081874 (PMC11351342; doi:10.3390/biomedicines12081874)
Supplement: Supplementary file 1 [file biomedicines-12-01874-s001.zip › Supplementary Figure/Figure S4/Singe SNP plot of MR analysis for TL to ubm-b-2037.pdf]

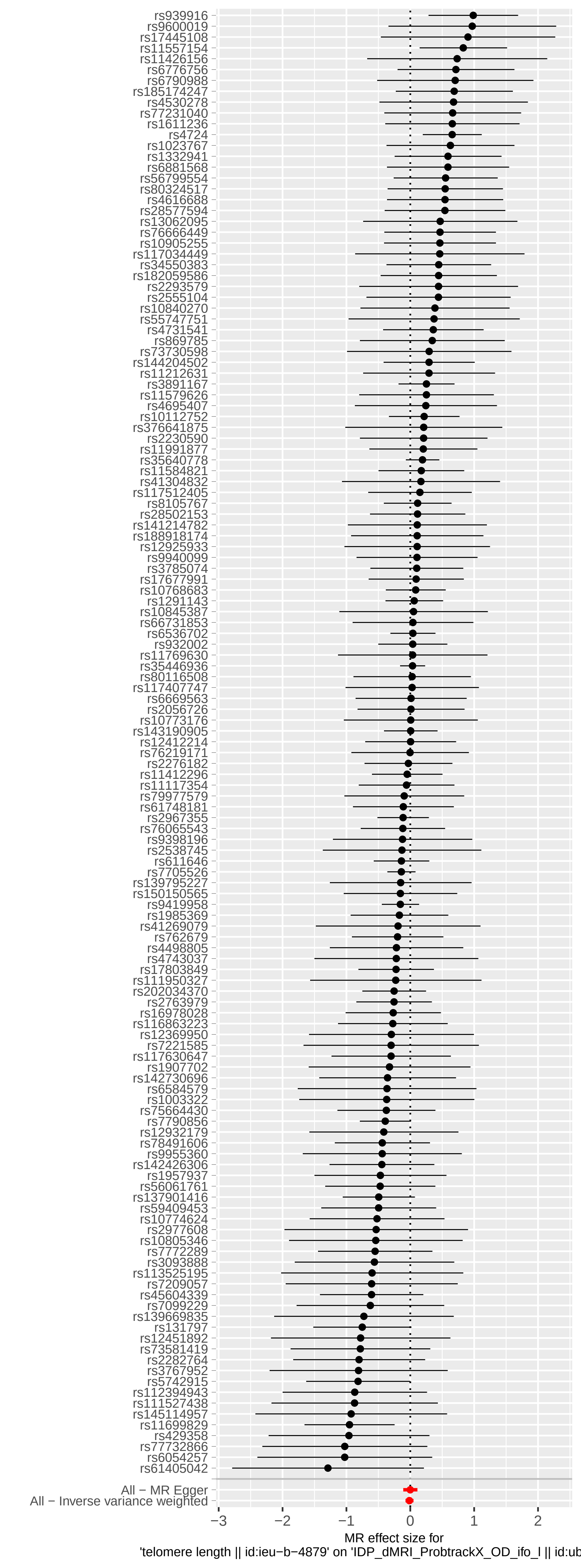
rs939916
rs9600019
rs17445108
rs11557154
rs11426156
rs6776756
rs6790988
rs185174247
rs4530278
rs77231040
rs1611236
rs4724
rs1023767
rs1332941
rs6881568
rs56799554
rs80324517
rs4616688
rs28577594
rs13062095
rs76666449
rs10905255
rs117034449
rs34550383
rs182059586
rs2293579
rs2555104
rs10840270
rs55747751
rs4731541
rs869785
rs73730598
rs144204502
rs11212631
rs3891167
rs11579626
rs4695407
rs10112752
rs376641875
rs2230590
rs11991877
rs35640778
rs11584821
rs41304832
rs117512405
rs8105767
rs28502153
rs141214782
rs188918174
rs12925933
rs9940099
rs3785074
rs17677991
rs10768683
rs1291143
rs10845387
rs66731853
rs6536702
rs932002
rs11769630
rs35446936
rs80116508
rs117407747
rs6669563
rs2056726
rs10773176
rs143190905
rs12412214
rs76219171
rs2276182
rs11412296
rs11117354
rs79977579
rs61748181
rs2967355
rs76065543
rs9398196
rs2538745
rs611646
rs7705526
rs139795227
rs150150565
rs9419958
rs1985369
rs41269079
rs762679
rs4498805
rs4743037
rs17803849
rs111950327
rs202034370
rs2763979
rs16978028
rs116863223
rs12369950
rs7221585
rs117630647
rs1907702
rs142730696
rs6584579
rs1003322
rs75664430
rs7790856
rs12932179
rs78491606
rs9955360
rs142426306
rs1957937
rs56061761
rs137901416
rs59409453
rs10774624
rs2977608
rs10805346
rs7772289
rs3093888
rs113525195
rs7209057
rs45604339
rs7099229
rs139669835
rs131797
rs12451892
rs73581419
rs2282764
rs3767952
rs5742915
rs112394943
rs111527438
rs145114957
rs11699829
rs429358
rs77732866
rs6054257
rs61405042
All − MR Egger
All − Inverse variance weighted
−3
−2
−1
0
1
2
MR effect size for
'telomere length || id:ieu−b−4879' on 'IDP_dMRI_ProbtrackX_OD_ifo_l || id:ub
